# The Effect of Laser Surface Treatment on the Bond Strength of Adhesive Materials to Primary Teeth: A Systematic Review

**DOI:** 10.3390/ma18225212

**Published:** 2025-11-18

**Authors:** Witold Świenc, Jan Kiryk, Mateusz Michalak, Zuzanna Majchrzak, Marzena Laszczyńska, Sylwia Kiryk, Natalia Grychowska-Gąsior, Izabela Nawrot-Hadzik, Jacek Matys, Maciej Dobrzyński

**Affiliations:** 1 Dental Surgery Department, Wroclaw Medical University, Krakowska 26, 50-425 Wroclaw, Poland; jan.kiryk@umw.edu.pl; 2 Medical Center of Innovation, Wroclaw Medical University, Krakowska 26, 50-425 Wroclaw, Poland; mateusz.michalak92@gmail.com (M.M.); zuzanna.h.nawrocka@gmail.com (Z.M.);; 3 Department of Pediatric Dentistry and Preclinical Dentistry, Wroclaw Medical University, Krakowska 26, 50-425 Wroclaw, Poland; s.roguzinska@gmail.com (S.K.); maciej.dobrzynski@umw.edu.pl (M.D.); 4 Department of Dental Prosthetics, Wroclaw Medical University, Krakowska 26, 50-425 Wroclaw, Poland; natgrychowska@gmail.com; 5 Department of Pharmaceutical Biology and Biotechnology, Faculty of Pharmacy, Wroclaw Medical University, 50-556 Wroclaw, Poland; izabela.nawrot-hadzik@umw.edu.pl

**Keywords:** adhesion, deciduous tooth, laser, milk tooth, primary tooth

## Abstract

This systematic review aimed to evaluate the effect of laser surface treatment on the bond strength of adhesive restorative materials to primary teeth. A comprehensive literature search was conducted in PubMed, Scopus, Embase, Web of Science and WorldCat up to July 2025 using the keywords primary teeth, deciduous teeth, milk teeth, laser, adhesion, bond strength. Twenty-six studies met the inclusion criteria, including 22 shear bond strength (SBS), three microtensile bond strength (µTBS) and one microshear bond strength (µSBS) investigations. Most studies evaluated erbium lasers (Er:YAG, Er,Cr:YSGG), while fewer assessed diode, Nd:YAG or KTP devices. In dentin, erbium lasers at low-to-moderate energy levels consistently produced smear-free, micro-retentive surfaces with open tubules and bond strengths comparable to bur-prepared controls. High-energy irradiation, however, frequently caused microcracks, tubule collapse and reduced adhesion. In enamel, phosphoric acid etching remained the most effective conditioning method, although combined laser–acid pretreatment often improved bonding of sealants and composites. Material-dependent effects were also evident: resin composites generally outperformed glass ionomers, hybrids and bioactive restorative materials. Phosphoric acid etching remains indispensable for enamel conditioning, while dentin benefits from carefully controlled erbium laser irradiation in combination with suitable adhesive systems.

## 1. Introduction

Oral health is a fundamental component of a child’s overall well-being. When left untreated, dental caries can progress to inflammation of the teeth and periodontium and may also contribute to systemic diseases [1,2]. Maintaining good oral hygiene in children, however, remains a considerable challenge for parents. Primary teeth differ from permanent teeth in their histological and morphological structure [3,4,5,6], which significantly influences both the onset and the progression rate of caries [4,5]. Because of their thinner enamel, larger pulp chamber and lower degree of mineralisation, primary teeth are more susceptible to demineralisation, cavity formation and subsequent pulp inflammation than permanent teeth. In contemporary restorative dentistry, adhesive materials are increasingly employed to restore primary dentition [7]. Nevertheless, the reduced mineralisation, higher density of dentinal tubules and lower density of intertubular dentin in primary teeth make achieving a durable restoration particularly challenging for clinicians [8,9,10]. Thus, establishing a strong and permanent bond between restorative materials and enamel or dentin is essential for treatment success [11].

The most common techniques for preparing tooth surfaces before restoration include mechanical preparation with rotary instruments and chemical etching with phosphoric acid [12,13]. However, these approaches are not without limitations [14]. Cutting enamel and dentin produces a smear layer that adversely affects the adhesive bond between restorative materials and dental tissues [15]. Even with the use of etching agents, complete removal of this layer is not possible [16]. Additional challenges arise from the specific histological features of primary teeth, which further compromise the retention of restorations [10]. Moreover, the physical stimuli generated during mechanical preparation are often perceived by children as unpleasant or threatening [17,18,19,20]. Despite the use of anaesthesia, limited cooperation and the relatively large pulp chamber with prominent pulp horns increase the risk of iatrogenic pulp injury [21]. Thus, achieving precise caries removal and creating optimal conditions for adhesion in primary teeth remain demanding tasks [22,23,24,25]. For this reason, safer and more effective alternatives—such as laser-based preparation techniques—are being investigated.

**Figure 1:**
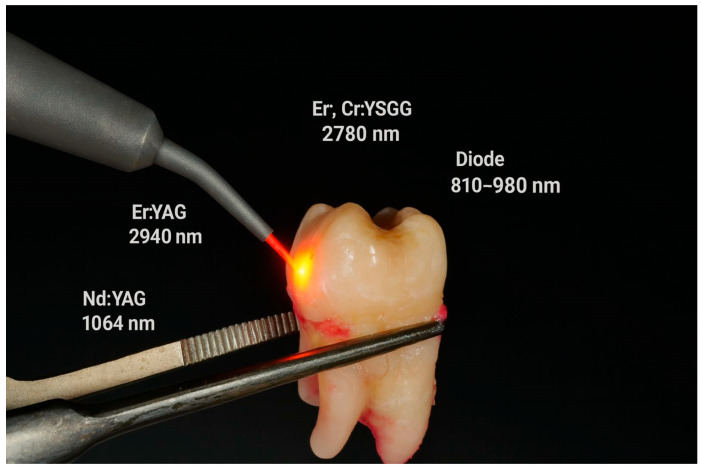
Lasers used for caries removal (Er:YAG, Er,Cr:YSGG) or for cavity disinfection (Nd:YAG, diode).

The introduction of laser technology into pediatric restorative dentistry has provided an alternative to conventional rotary instrumentation [26,27]. Among the most widely studied systems, Er:YAG (2940 nm) and Er,Cr:YSGG (2780 nm) lasers demonstrate strong absorption in water and hydroxyapatite, allowing selective ablation of enamel and dentin with minimal thermal damage [28,29,30,31]. Their application results in smear layer removal, exposure of dentinal tubules and the formation of micro-retentive surface patterns that may facilitate adhesive penetration [28,29,32,33]. By contrast, Nd:YAG and diode lasers, though less effective in hard-tissue ablation, have been investigated for their ability to modify dentin surface energy and collagen structure, which may also influence bonding [29,32]. Evidence suggests that the adhesive strength achieved depends more on laser-induced surface characteristics than on the cutting effect itself, highlighting the importance of morphological evaluation (e.g., SEM analysis) in assessing clinical relevance [27,28]. In addition to these biological benefits, laser systems reduce vibration and noise, thereby improving treatment tolerance and acceptance in pediatric patients [26,27,29]. (Figure 1).

Available studies on adhesion in laser-treated primary teeth have reported heterogeneous outcomes. Several in vitro experiments demonstrated that Er:YAG irradiation can enhance shear bond strength by eliminating the smear layer and opening dentinal tubules, thereby facilitating adhesive penetration [28,34]. Conversely, other investigations found weaker or unchanged bond values when suboptimal energy settings were applied, indicating that outcomes depend strongly on the technique and laser parameters used [28,35]. Clinical trials have shown comparable retention of composite restorations in cavities prepared with either Er:YAG lasers or conventional burs, suggesting that both methods may perform similarly under certain conditions [35,36]. Systematic reviews and meta-analyses further confirm this variability, with some reporting improved adhesion and others showing no significant benefit or even reduced bond strength [28,34,35]. These inconsistencies underscore the need to standardize laser protocols and to conduct long-term studies specifically focused on primary dentition [27,29,36,37,38].

This review aims to evaluate the effect of laser surface treatment on the bond strength of adhesive materials to primary teeth. To our knowledge, it represents the first systematic analysis to compile evidence from multiple studies assessing whether laser conditioning of primary teeth surfaces significantly improves the retention of adhesive restorations. The findings are intended to support clinicians in developing optimized treatment protocols for primary dentition. By reducing the discomfort associated with conventional preparation methods, laser treatment may not only enhance patient cooperation but also minimize the risk of tissue damage while creating conditions favorable for strong adhesive bonding.

## 2. Materials and Methods

### 2.1. Focused Question

This systematic review was structured according to the PICO framework [39] as follows: In primary teeth (Population), does surface preparation with a laser (Intervention) influence shear bond strength (Outcome) compared with traditional preparation methods (Comparison)?

### 2.2. Protocol

**Figure 2:**
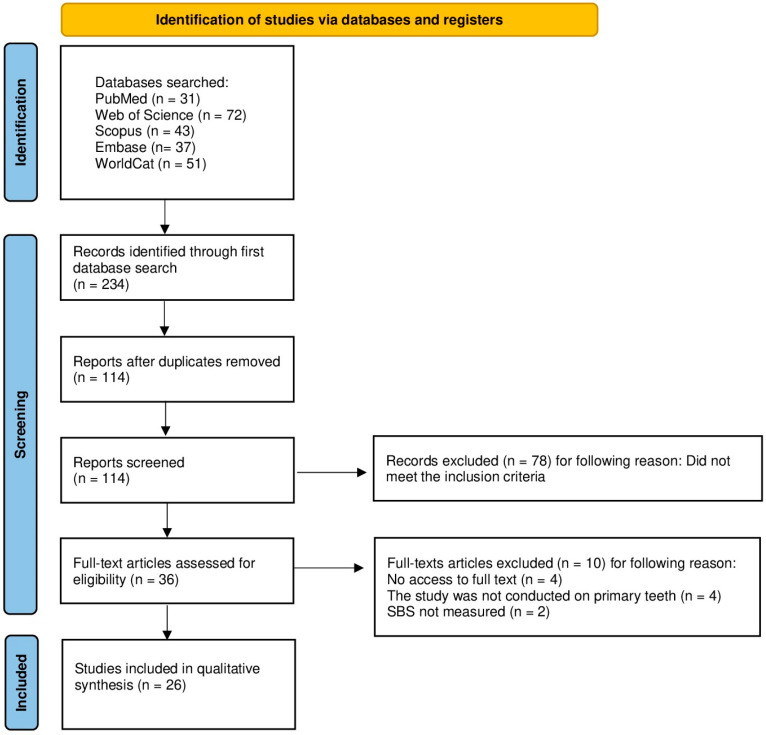
PRISMA 2020 Flow diagram.

The procedure for selecting articles was organized in accordance with the PRISMA guidelines (Figure 2) [40,41,42,43,44]. Details of the protocol, including registration of the systematic review, are available in the Open Science Framework at the following link: https://osf.io/zu6cr (accessed on 27 September 2025).

### 2.3. Eligibility Criteria

The researchers agreed to include only the articles that met the following criteria:

- Original research articles;
- Laser pretreatment;
- Using all kind of composite materials;
- SBS evaluation studies;
- In vitro studies;
- Examinations performed on primary teeth;
- Studies in English;
- Full-text articles.

The exclusion criteria the reviewers agreed upon were as follows:

- No laser pretreatment;
- No SBS evaluation;
- Studies conducted on permanent teeth;
- Non-English papers;
- Systematic review articles;
- Review articles;
- No full-text accessible;
- Duplicated publications.

No restrictions were applied with regard to the year of publication.

### 2.4. Information Sources, Search Strategy, and Study Selection

A comprehensive literature search was carried out in July 2025 across four major databases: PubMed, Scopus, Embase and Web of Science (WoS). The search strategy was designed to identify studies that fulfilled the predefined inclusion criteria. To ensure accuracy, the queries combined terms related to primary dentition, laser application and bond strength or adhesion. The results were restricted to titles, abstracts and keywords. Only articles available in full text and consistent with the eligibility criteria were considered for further analysis.

The exact search strategies used in each database were as follows:

PubMed: (“primary teeth” OR “deciduous teeth” OR “milk teeth”) AND (“laser”) AND (“bond strength” OR “adhesion”).

Scopus: TITLE-ABS-KEY (“primary teeth” OR “deciduous teeth” OR “milk teeth”) AND TITLE-ABS-KEY (“laser”) AND TITLE-ABS-KEY (“bond strength” OR “adhesion”).

Web of Science (WoS)**:** TS = (“primary teeth” OR “deciduous teeth” OR “milk teeth”) AND TS = (“laser”) AND TS = (“bond strength” OR “adhesion”).

Embase: (‘primary teeth’ OR ‘deciduous teeth’ OR ‘milk teeth’) AND (laser) AND (‘bond strength’ OR adhesion).

WorldCat: (“primary teeth” OR “deciduous teeth” OR “milk teeth”) AND (“laser”) AND (“bond strength” OR “adhesion”).

### 2.5. Data Collection and Data Items

Six independent reviewers (J.K., Z.N., M.M., M.L., W.Ś. and S.K.) screened and selected the studies that fulfilled the inclusion criteria. The relevant information extracted from each article was organized and recorded in a standardized Excel spreadsheet.

### 2.6. Assessing Risk of Bias in Individual Studies

During the initial screening stage, the reviewers independently assessed the titles and abstracts of all identified studies to minimize the risk of selection bias. The degree of agreement between reviewers was measured using Cohen’s κ statistic [45]. Any discrepancies regarding study inclusion or exclusion were resolved through discussion until a consensus was reached.

### 2.7. Quality Assessment

Two independent reviewers (J.M. and M.D.), blinded to each other’s assessments, evaluated the methodological quality of all included studies using the *Critical Appraisal Checklist for Quasi-Experimental (Non-Randomized) Studies* developed by the Joanna Briggs Institute (JBI), an international center for evidence-based practice. The JBI checklist is designed to assess the risk of bias and overall reliability of research findings. Each version of the checklist is tailored to a specific study design and focuses on methodological rigor, internal validity, and the clarity of reporting. The quasi-experimental version applied in this review includes nine domains covering key aspects such as participant selection, comparability of groups, intervention integrity, outcome measurement, and statistical analysis.

Q1 Is it clear in the study what is the ‘cause’ and what is the ‘effect’?
Q2 Were the participants included in any similar comparisons?
Q3 Were the participants included in any comparisons receiving similar treatment/care, other than the exposure or intervention of interest?
Q4 Was there a control group?
Q5 Were there multiple measurements of the outcome both before and after the intervention/exposure?
Q6 Was a follow up completed, and if not, were differences between groups in terms of their follow up adequately described and analyzed?
Q7 Were the in vitro results of participants included in any comparisons measured in the same way?
Q8 Were the outcomes measured in a reliable way?
Q9 Was an appropriate statistical analysis used?

Each item on the checklist was rated using one of four possible responses: “yes,” “no,” “unclear” or “not applicable.” Any discrepancies in scoring were resolved through discussion until a consensus was reached. Inter-rater reliability was assessed using Cohen’s kappa coefficient, calculated in MedCalc software (version 23.1.7; MedCalc Software Ltd., Ostend, Belgium). The resulting κ value of 0.81 (*p* < 0.001) indicated an excellent level of agreement and consistency between the two reviewers.

## 3. Results

### 3.1. Study Selection

The database search initially identified 234 articles that appeared to meet the inclusion criteria. After removing duplicates, 114 unique records were retained. Screening of titles and abstracts led to the exclusion of 78 papers that did not satisfy the eligibility requirements. A full-text review of the remaining 36 articles resulted in the exclusion of 10 studies: four due to lack of full-text access, four because they were conducted on permanent teeth and two for not reporting shear bond strength (SBS). Consequently, 26 studies fulfilled all the predefined criteria and were included in this systematic review. These investigations focused on the influence of laser surface treatment on the bond strength of adhesive materials to primary teeth. A quantitative meta-analysis was not performed due to the substantial heterogeneity among the included studies. The identified investigations differed in laser types (Er:YAG, Er,Cr:YSGG, diode, Nd:YAG), irradiation parameters (energy, power, frequency, and distance), substrate types (enamel vs. dentin), adhesive systems (etch-and-rinse, self-etch, or self-adhering), and bond strength testing protocols (SBS, µSBS, µTBS).

### 3.2. General Characteristics of the Included Studies

The 26 studies included in this review investigated the effect of laser surface treatment on the bond strength of adhesive materials to primary teeth, applying a wide range of laser systems and operating protocols. Erbium-based lasers were the most frequently applied: Er:YAG was used in 15 studies [46,47,48,49,50,51,52,53,54,55,56,57,58,59,60], while Er,Cr:YSGG was employed in 9 investigations [47,61,62,63,64,65,66,67,68]. Other laser types appeared less often, including diode [69,70] and KTP [71], which were also compared to the Er:YAG laser. Reported laser parameters included wavelength, pulse energy, frequency, spot size, irradiation distance, exposure time and cooling conditions. Several studies compared different parameter settings within the same device to assess dose–response effects, including Er:YAG energy and frequency variations [48,51,53,54,57,59] and Er,Cr:YSGG power outputs [63,65].

The tested substrates were enamel in 11 studies [46,47,52,55,56,61,63,64,65,68,69] and dentin in 18 studies [47,48,49,50,51,52,53,54,57,58,59,60,62,66,67,69,70,71]. Four studies investigated both tissues within the same experiment [47,49,52,69].

A wide range of restorative materials was evaluated. Etch-and-rinse adhesives were applied in eight studies [46,48,49,51,56,57,59,60], while self-etch adhesives were tested in four [50,51,53,54]. Resin composites were the most frequently assessed material, used in ten studies [49,51,52,57,58,59,60,66,67,70]. Bioactive restorative materials or glass hybrids were included in two investigations [69,70]. Pit-and-fissure sealants were evaluated in five studies [46,55,61,63,65]. Two studies focused on self-adhering flowable composites [52,68]. Additionally, some studies incorporated adjunctive surface pretreatments such as CPP-ACP, sodium hypochlorite (NaOCl), or chlorhexidine (CHX) [60,61,71].

Shear bond strength (SBS) was the most frequently employed testing method, reported in 22 studies. In addition, three studies assessed microtensile bond strength (µTBS) [53,54,63] and one used a micro-shear protocol (µSBS) [68]. In dentin, appropriately selected erbium-laser settings (e.g., 50–200 mJ for Er:YAG; 2.5–3.5 W for Er,Cr:YSGG) typically produced smear-free, micro-retentive surfaces with open tubules, yielding bond strengths comparable to bur-prepared controls [48,50,51,53,57,60]. By contrast, excessive energy levels or high repetition rates often led to microcracks, thermal alterations and reduced bond values [48,54]. In enamel, laser conditioning alone frequently resulted in lower SBS compared with conventional phosphoric acid etching [46,49,55,56,65]. However, combining laser irradiation with subsequent acid etching generally enhanced adhesion, particularly for sealants and resin composites [46,55,56,63,65].

Most of the included studies complemented bond strength testing with morphological evaluations, primarily by scanning electron microscopy (SEM) and in some cases by EDX [67]. A total of 13 investigations reported surface analyses [48,49,50,51,52,53,54,57,58,60,67,70,71]. These consistently showed that erbium laser treatment produced smear-free, micro-retentive surfaces with open dentinal tubules and irregular, scaly morphologies, in contrast to the smoother, smear-covered surfaces observed after bur preparation. Depending on the irradiation parameters, additional features such as remelted zones, subsurface cracking, microporosity and disrupted enamel prisms were observed [48,54,67]. Failure-mode analyses further supported these findings: mixed failures predominated in groups with higher bond strength [48,52,53,55], whereas adhesive failures were more common when excessive laser energy or insufficient surface conditioning compromised adhesion [46,49,54,65].

#### 3.2.1. Shear Bond Strength

Shear bond strength (SBS) was the most frequently reported outcome, assessed in 22 studies [46,47,48,49,50,51,52,55,56,57,59,60,61,62,63,64,65,66,68,69,70,71]. Reported SBS values varied according to laser type, energy settings, substrate and adhesive material. In dentin, erbium lasers operated at moderate parameters (50–200 mJ for Er:YAG; 2.5–3.5 W for Er,Cr:YSGG) generally produced smear-free, micro-retentive surfaces with open tubules, achieving bond strengths comparable to bur-prepared controls [47,48,50,51,52,57,59,60]. Conversely, higher energy levels or repetition rates (≥250–300 mJ or >20 Hz) frequently resulted in significant reductions in SBS, accompanied by microcracks, loss of tubular structure or thermal damage [48,51,54,56,57].

For enamel, laser conditioning alone often yielded lower SBS compared with conventional phosphoric acid etching [46,49,55,56,65]. However, combining laser pretreatment with acid etching enhanced adhesion, particularly for pit-and-fissure sealants and resin composites [46,55,56,63,65]. Material-specific differences were also observed: bioactive restorative materials and glass ionomer/hybrid cements consistently showed reduced SBS compared with resin composites, regardless of surface conditioning protocol [62,66,69,70]. Some investigations further employed micro-shear bond strength (µSBS) testing as a variation of SBS [65,68,69].

#### 3.2.2. Microtensile Bond Strength

Microtensile bond strength (µTBS) was assessed in three studies [53,54,63], while one additional study reported micro-shear bond strength (µSBS) [68]. In all cases, dentin surfaces treated with Er:YAG or Er,Cr:YSGG lasers exhibited open tubules, absence of smear layer and irregular scaly morphologies. Flury et al. tested a broad range of Er:YAG parameters (50–400 mJ, 20–35 Hz) and found no significant differences in µTBS between laser- and bur-prepared dentin (22.2–26.1 MPa vs. 24.8 MPa; *p* = 0.394) [53]. Yildiz et al. reported significantly reduced µTBS in laser-prepared dentin compared with bur or Carisolv preparation, both for etch-and-rinse (15.7 ± 5.9 MPa vs. 20.8 ± 5.6 MPa) and self-etch adhesives (16.5 ± 4.9 MPa vs. 21.1 ± 5.2 MPa) [54]. AlHumaid et al. demonstrated that laser conditioning at 2.5 W resulted in lower µTBS, whereas 3.5 W achieved bond strengths comparable to acid etching [63]. Kiomarsi et al., using a µSBS protocol, showed that laser pretreatment improved bonding of self-adhering flowables but had no significant effect on conventional composites [68]. Overall, these findings indicate that the effect of laser pretreatment on tensile bonding depends strongly on energy settings and adhesive type.

#### 3.2.3. Morphological Analysis

Morphological evaluations were reported in 13 studies, mainly using scanning electron microscopy (SEM), with one study also employing EDX [47,48,49,50,51,52,53,54,57,60,62,67,69]. Across these investigations, erbium laser irradiation consistently removed the smear layer, exposed dentinal tubules and produced irregular, micro-retentive surfaces, in contrast to the smoother, smear-covered morphology observed after bur preparation [47,48,49,50,51,53,54,57,62]. Depending on laser parameters, additional features were documented, including remelted zones, recrystallized enamel prisms, microporosities and microcracks [46,48,54,56,57,65,67]. In enamel, high-energy Er:YAG irradiation sometimes caused subsurface cracking and irregular etching patterns [4,21,22], whereas in dentin, excessive power or frequency led to tubule collapse and localized carbonization [48,54,57].

Failure-mode analyses supported these observations: mixed or cohesive failures predominated in groups with higher bond strength and favorable surface morphology [47,48,50,52,53,54,55,69], while adhesive failures were most frequent when laser settings induced structural damage or insufficient micromechanical retention [46,49,55,56,65,69] (see Table 1).

### 3.3. Main Study Outcomes

The synthesis of the 26 included studies demonstrates that laser surface treatment substantially modifies the structure of primary tooth tissues and influences the bonding performance of adhesive materials. Among the reviewed investigations, 22 assessed shear bond strength (SBS/µSBS) [46,47,48,49,50,51,55,56,57,59,60,61,62,63,64,65,66] three focused on microtensile bond strength (µTBS) [53,54,63], and one applied a µSBS protocol [68] (see Table 2). Collectively, the findings confirm that laser parameters, substrate type, and restorative material critically determine the quality of adhesion.

Erbium lasers (Er:YAG and Er,Cr:YSGG) operated at low-to-moderate energy levels consistently produced smear-free, micro-retentive surfaces with open dentinal tubules, yielding bond strengths comparable to or exceeding those obtained with conventional bur preparation [47,48,50,52,53,55,57,59,60]. Although the literature lacks universally accepted thresholds for clinically adequate SBS values in primary teeth, most studies considered the range of 17–20 MPa as acceptable. For instance, Wang et al. reported optimal adhesion with Er:YAG parameters between 100 and 150 mJ [48], whereas Bahrololoomi et al. observed no significant differences between laser and bur conditioning when moderate energy was used [49]. However, when energy or repetition rates exceeded 250–300 mJ or 20 Hz, surface damage became evident. Under such conditions, microcracks, thermal alteration, or tubule collapse often led to a measurable decline in bond strength [48,51,54,56,57]. Similarly, Yildiz et al. found that over-irradiated dentin produced lower µTBS values than those obtained after bur or Carisolv preparation, illustrating the detrimental effects of excessive laser exposure [54].

Differences between enamel and dentin conditioning were also evident. In enamel, phosphoric acid etching remained the most effective and reliable technique, consistently outperforming laser treatment alone [46,49,55,56,65]. Nevertheless, a synergistic effect was frequently observed when erbium laser irradiation was followed by acid etching, particularly for pit-and-fissure sealants and resin composites [46,55,56,63,65]. In dentin, optimized laser parameters promoted the removal of the smear layer and opened dentinal tubules, resulting in improved adhesion compared with inadequately adjusted settings [53,55,56,58,62,65].

The type of restorative material also played a crucial role in bonding outcomes. Resin composites generally achieved the highest bond strengths regardless of the conditioning method [54,56,62,64,65]. In contrast, bioactive restorative materials, glass ionomers, and glass hybrids consistently demonstrated inferior adhesion, yielding significantly lower SBS values across most studies [62,66,69,70]. Both Kotb and Bolukbasi reported reduced adhesion of bioactive or hybrid materials compared with conventional composites [69,70]. Investigations involving self-adhering flowable composites produced mixed results: while Memarpour showed that Er:YAG pretreatment enhanced Vertise Flow adhesion [57], Kiomarsi found that laser conditioning improved µSBS in self-adhering flowables but had no measurable effect on traditional composites [68].

Several studies also confirmed a dose–response relationship between laser parameters and adhesive performance. AlHumaid et al. demonstrated that increasing Er,Cr:YSGG power from 2.5 W to 3.5 W significantly improved µTBS, particularly when combined with phosphoric acid etching [63]. Likewise, Sungurtekin-Ekci and Oztas observed that higher power (3.5 W) produced better results than lower power (2.5 W), with acid conditioning further enhancing the bond [65]. Conversely, Paryab et al. showed that dentin conditioning with Er:YAG at 300–400 mJ reduced SBS compared to bur-prepared samples [51], and Moghini et al. reported progressive deterioration in both adhesion and surface morphology as output energy increased from 60 to 100 mJ [57].

Morphological analyses performed using SEM and complementary techniques (EDX, AFM) consistently supported the mechanical findings. Favorable bonding outcomes were associated with laser-treated surfaces that exhibited open dentinal tubules, irregular scaly microtopography, and absence of smear layer [47,48,49,50,51,52,53,54,57,59,62,67]. These structural features correlated with higher SBS or µTBS values and a predominance of mixed or cohesive failure modes [52,53,55,57,58,59,60]. In contrast, excessive irradiation produced adverse morphological effects—microcracks, melted areas, and tubule collapse—which coincided with lower bond strengths and mainly adhesive failures [48,51,52,56,57,67,69].

Across the included studies, both Er:YAG and Er,Cr:YSGG lasers achieved the most favorable adhesion outcomes when operated at low-to-moderate energy and power levels with adequate air–water cooling. Specifically, energies of 120–200 mJ (≈1.2–2.0 W) for enamel and 80–150 mJ (≈0.8–1.5 W) for dentin using the Er:YAG laser were consistently associated with micro-retentive, smear-free surfaces and bond strengths comparable to or exceeding those produced by bur preparation. For Er,Cr:YSGG lasers, optimal performance was observed at 2.5–3.5 W (≈125–175 mJ per pulse at 20 Hz), particularly when combined with phosphoric acid etching. Excessive energy input (>250–300 mJ for Er:YAG or >4 W for Er,Cr:YSGG) resulted in microcracks and thermal alterations, leading to a measurable reduction in bond strength. The findings indicate that precise adjustment of laser parameters is essential to maximize adhesion and prevent structural damage, providing a practical reference for clinicians using erbium lasers in pediatric restorative procedures (see Table 3).

### 3.4. Quality Assessment

All included studies were appraised using the JBI Critical Appraisal Checklist. Overall, the methodological quality was high, with most studies showing a low risk of bias. A total of 24 studies were rated as low risk of bias [46,47,48,49,50,51,52,53,54,55,56,57,59,60,61,62,63,64,65,66,68,69,70,71], with 2 studies being rated as moderate risk of bias due to shortcomings in statistical analysis: Felemban [58] and Oznurhan [67]. Importantly, no study was judged to be at high risk of bias. The main limitation identified across all studies was the absence of multiple outcome measurements before and after the intervention. Nevertheless, outcome measures were generally consistent and reliable, control groups were present and follow-up was either complete or adequately analyzed. (See Table 4). The quality assessment process followed the PRISMA 2020 Checklist [72], as detailed in the Supplementary Materials (Table S1).

## 4. Discussion

The present systematic review evaluated the influence of laser surface treatment on the bond strength of restorative materials to primary teeth. Overall, the findings revealed considerable variability in outcomes, largely determined by the type of laser, its operating parameters, and the specific clinical protocol applied. In several studies, the adhesive performance of laser-conditioned surfaces was comparable to that achieved with conventional techniques. For example, Er,Cr:YSGG irradiation at 3.5 W produced shear bond strength values of composite resins similar to those obtained with phosphoric acid etching [63]. However, when lasers were used as the sole conditioning method—particularly Er:YAG on enamel—the resulting adhesion was often significantly lower, underscoring that laser irradiation alone remains insufficient as a substitute for acid etching [51]. A consistent trend across studies indicated that combining erbium laser pretreatment with phosphoric acid etching generally improved adhesion compared to laser conditioning alone, although the results seldom exceeded those achieved with conventional acid etching [52]. AlHumaid et al. demonstrated a clear dose-dependent effect, where Er,Cr:YSGG operated at 3.5 W achieved bond strengths comparable to acid etching, while the lower 2.5 W setting produced markedly weaker adhesion [63]. Similarly, Wang et al. showed that Er:YAG irradiation within the moderate range of 50–200 mJ and 5–20 Hz enhanced dentin bonding, whereas higher energies caused microstructural alterations and reduced adhesion [48]. These findings collectively emphasize that the clinical success of laser conditioning depends critically on optimizing operating parameters.

The type of laser and its precise calibration directly influence the resulting micromorphology. Er:YAG irradiation without subsequent acid conditioning consistently yielded the lowest bond strength values, while the addition of phosphoric acid substantially improved adhesion [46]. For Er,Cr:YSGG, increasing power output from 2.5 W to 3.5 W eliminated much of the reduction in bond strength, producing results equivalent to those of acid-etched enamel [65]. Conversely, diode laser irradiation at 980 nm significantly decreased the bond strength of both Activa Bioactive and composite materials, suggesting an adverse influence on adhesive performance [69]. In dentin, the sensitivity to parameter adjustment was even more pronounced. Er:YAG irradiation at 50–200 mJ and 5–20 Hz generated favorable micromorphology characterized by open tubules and absence of a smear layer, thereby enhancing adhesion [48]. Beyond these settings, deleterious changes such as tubule collapse, charring, and microcracking were observed, leading to diminished bonding efficacy. Moreover, the irradiation distance influenced the results: at 20 mm, SBS values were the highest, whereas shorter distances weakened adhesion, particularly when laser conditioning was combined with acid etching [59]. These findings highlight the complexity of controlling multiple interacting variables—wavelength, power, frequency, fluence, distance, exposure time, and spot size—and the urgent need for standardized, reproducible clinical protocols tailored to primary dentition.

Adhesive performance was also strongly affected by the choice of bonding system and restorative material. Koyuturk et al. demonstrated that the self-etch adhesive Clearfil S3 produced superior bonding to laser-prepared dentin compared with Xeno V, indicating that compatibility between the adhesive and the laser-modified surface is critical [50]. Memarpour similarly found that Er:YAG pretreatment enhanced the bond strength of the self-adhering flowable composite Vertise Flow on enamel but not on dentin, where performance remained similar to that achieved with conventional SiC preparation [52]. Kiomarsi further confirmed the material-dependent nature of this effect, reporting that laser conditioning improved microshear bond strength for self-adhering flowables but not for traditional composites [68]. For pit-and-fissure sealants, Er,Cr:YSGG irradiation at 3.5 W yielded adhesion comparable to phosphoric acid etching [68]. Laser conditioning also appeared beneficial for certain glass ionomer cements: Chikkanarasaiah and Hrishida found that Er,Cr:YSGG pretreatment provided stronger bonding for Fuji IX than conventional 10% poly (acrylic acid) conditioning [3]. In contrast, diode laser treatment reduced the bond strength of the bioactive material Activa [69]. Collectively, these findings suggest that the success of laser pretreatment depends not only on laser parameters but also on the interaction between the modified substrate and the specific adhesive–material system used.

When compared directly with traditional methods, laser conditioning rarely achieved superior results. Babu et al. observed that the highest bond strengths consistently occurred after bur preparation combined with phosphoric acid etching, whereas laser pretreatment alone produced substantially lower values. Even when followed by acid etching, laser-treated surfaces did not fully match the adhesion achieved with conventional techniques [47]. Bahrololoomi et al. reached similar conclusions, showing that bur and acid etching remained the most effective combination [49]. In caries removal studies, Yildiz et al. reported that dentin prepared with Er:YAG exhibited lower bond strength than surfaces treated with burs or Carisolv [54]. Interestingly, clinical follow-up by Felembam revealed that after 12 months, no significant differences were observed in the interfacial quality between bur- and laser-prepared cavities [63]. This discrepancy underscores the potential divergence between in vitro mechanical data and in vivo clinical outcomes. While bond strength tests provide valuable information about initial adhesion, patient comfort, tolerance, and the long-term adaptation of restorations play equally important roles in pediatric practice. The primary clinical advantage of laser preparation lies in improved patient acceptance—particularly among children—due to reduced vibration, noise, and anesthetic requirements. However, these benefits must be weighed against the disadvantages, including the high cost of equipment, procedural complexity, and the risk of morphological damage when inappropriate energy levels are used.

The collective evidence from the reviewed studies is limited by several methodological constraints. Many investigations involved small sample sizes—for instance, Malekafzali [64] and Oznurhan [67] included only 20 specimens—thus restricting the statistical robustness of their conclusions. Furthermore, the wide heterogeneity in study design, laser type, power settings, and irradiation distances complicates cross-comparison and meta-analysis [63,64,69]. Long-term data are scarce; Borsatto et al. [55] reported significant reductions in bond strength after six months of aging under simulated oral conditions, raising concerns about the durability of laser-conditioned adhesion. Moreover, thermomechanical alterations such as localized heating, dentinal dehydration, or microcrack formation may further compromise the long-term integrity of the adhesive interface, although such effects remain insufficiently studied in primary dentition. Several experiments also excluded caries-affected dentin, which is clinically unavoidable [55], and those that included it demonstrated less favorable outcomes for laser-prepared surfaces than for bur or Carisolv methods [54]. Additionally, inherent structural differences between primary and permanent teeth—including reduced mineralization, increased water content, and distinct tubule architecture—further challenge the extrapolation of findings [60]. From a clinical perspective, current evidence supports the use of lasers as valuable adjuncts rather than replacements for conventional surface conditioning. However, cost-effectiveness remains a relevant limiting factor; despite advantages such as improved patient comfort and reduced need for anesthesia, the high cost of devices and lack of standardized parameters currently restrict their routine application in pediatric restorative dentistry.

## 5. Conclusions

This systematic review demonstrated that laser surface conditioning—particularly with erbium-based devices (Er:YAG and Er,Cr:YSGG)—served as a valuable adjunct, and in selected cases, a viable alternative to conventional preparation of primary teeth prior to adhesive restorative procedures. When operated within controlled, low-to-moderate energy ranges, erbium lasers consistently produced favorable micro-morphological changes, including the absence of smear layers, exposure of dentinal tubules, and the formation of irregular micro-retentive surfaces. These characteristics translated into bond strengths comparable to, and occasionally exceeding, those achieved with traditional bur preparation. Conversely, high-energy or high-frequency settings often induced adverse morphological effects—such as microcracks, thermal damage, or collapse of dentinal tubules—which resulted in significantly reduced adhesion. For enamel, the evidence remained less consistent: laser conditioning alone was insufficient to replace phosphoric acid etching, yet combined erbium laser and acid etching protocols frequently produced enhanced outcomes, particularly for resin composites and pit-and-fissure sealants. In dentin, controlled erbium irradiation created surfaces that were highly suitable for bonding, while diode and Nd:YAG lasers—though less extensively studied—showed variable or inferior performance. Material-specific trends were also evident: resin composites consistently achieved higher bond strengths than glass ionomers, glass hybrids, or bioactive restorative materials, regardless of surface treatment. Future research should focus on well-designed randomized clinical trials employing standardized laser protocols under clinically relevant conditions (e.g., contamination, thermomechanical loading, and long-term aging). Greater attention to adhesive stability over time, and to interactions with new-generation adhesives and bioactive materials, was considered essential to further clarify the clinical role of laser conditioning in pediatric restorative dentistry.

## Figures and Tables

**Table 1:** General characteristics of studies.

Study	Aim of the Study	Material and Methods	Results	Conclusions
Ersan [61]	To assess how Er,Cr:YSGG laser disinfection, CPP-ACP paste application and NaOCl (alone and combined) affect SBS to primary teeth enamel.	Several pre-application protocols (none, Er,Cr:YSGG, Er,Cr:YSGG+CPP-ACP, NaOCl, NaOCl+CPP-ACP, CPP-ACP) were performed on the polished buccal surfaces of primary teeth, after which a sealant was applied and the SBS and fracture patterns were examined.	The lowest SBS was obtained after NaOCl (alone or with CPP-ACP), with a predominance of adhesive fractures; differences between the other groups were not significant.	Er,Cr:YSGG disinfection and CPP-ACP application may be acceptable alternatives before sealant, but NaOCl weakens the bond.
Chikkanarasaiah and Hrishida [62]	Comparison of SBS glass ionomer cement (Fuji IX) to primary dentin after standard conditioning with 10% poly (acrylic acid) vs. after conditioning with Er,Cr:YSGG laser.	30 primary teeth were prepared for two protocols (PA 10% or Er,Cr:YSGG 0.5 W/25 mJ), GIC restorations, thermocycling and SBS test were performed.	Er,Cr:YSGG conditioning increased SBS GIC relative to standard poly (acrylic acid) conditioning.	Laser dentin conditioning may improve the adhesion of glass ionomer cement in primary teeth.
Borsatto [46]	To assess whether enamel conditioning with an Er:YAG laser affects tensile bond strength (TBS) in primary teeth	Thirty primary molars were randomly divided into 3 groups (acid, Er:YAG, Er:YAG+acid), enamel was flattened, sealant was applied, samples were subjected to thermocycling and TBS test.	TBS was similar for “acid” (10.80 ± 3.28 MPa) and “Er:YAG+acid” (12.85 ± 2.14 MPa) and significantly lower for “Er:YAG alone” (4.17 ± 2.31 MPa).	Er:YAG irradiation alone does not provide retention—subsequent etching with phosphoric acid is necessary.
Kotb [69]	To assess whether diode laser irradiation (980 nm, 1 W) changes the micro shear bond strength (µSBS) of Activa Bioactive material and composite on enamel and dentin of primary teeth.	104 primary molars were divided into groups of “laser/no laser” and enamel/dentin and Activa/composite substrates; etching/universal adhesive, laser (or no laser), 500 thermal cycles, and µSBS testing were performed.	The laser significantly reduced the µSBS of Activa (on both enamel and dentin) and did not improve the µSBS of the composite.	Diode laser irradiation before polymerization does not increase the adhesion of the composite in primary teeth and impairs the bonding of Activa.
Babu [47]	Compare the shear bond strength (SBS) of the composite after cavity preparation in primary teeth using the classical method and the Er:YAG and Er,Cr:YSGG lasers.	100 primary molars were randomly assigned to 5 conditioning regimens (phosphate, Er:YAG+acid, Er,Cr:YSGG+acid, Er:YAG alone, Er,Cr:YSGG alone), followed by composite restorations, thermocycling, and SBS testing.	The highest SBS was obtained after classical etching; laser etching alone gave significantly lower values, which were improved by the addition of acid etching.	Phosphoric acid etching remains the most effective, and adding acid to laser-prepared surfaces improves bond strength.
AlHumaid [63]	To evaluate how different Er,Cr:YSGG laser powers affect µTBS to primary enamel.	50 first primary molars were divided into 5 groups: 3.5 W + acid, 2.5 W + acid, 3.5 W without acid, 2.5 W without acid, acid only; sealant application and µTBS test were performed.	All groups except “2.5 W acid-free” had similar μTBS; 2.5 W ablation alone gave significantly lower values (9.66 MPa).	3.5 W Er,Cr:YSGG used solo provides µTBS comparable to acid, suggesting that with appropriate power the laser can effectively condition enamel for sealant.
Wang [48]	To investigate how Er:YAG laser dentin pretreatment parameters affect dentin morphology and composite bond strength in primary teeth.	Dentin plates from primary molars were subjected to: no treatment, acid etching, Er:YAG etching with different energy (50–300 mJ) or frequency (5–30 Hz); SEM and SBS were assessed after thermocycling.	In the range of 50–200 mJ and 5–20 Hz, the laser opened the tubules without lamellar rupture and increased SBS; outside this range, ruptures/intertubular damage were observed and no further improvement was observed.	Properly selected Er:YAG parameters can improve bonding to dentin in primary teeth by opening the tubules without creating a smear layer.
Bahrololoomi [49]	Compare the effect of bur vs. Er:YAG laser preparation (with/without etching) on SBS composite for enamel and dentin of primary teeth.	75 primary molars were cut mesiodistally (150 samples) and in each tissue (enamel/dentin) three schemes were performed: drill+acid, Er:YAG+acid, Er:YAG only; Single Bond and Z250, thermocycling and SBS test were used.	The highest SBS was obtained with drill+acid, lower with Er:YAG+acid, and lowest with Er:YAG alone; enamel generally had a higher SBS than dentin.	Laser preparation generates lower SBS than mechanical preparation, especially without additional etching.
Malekafzali [64]	Comparison of the shear bond strength of composite with primary tooth enamel after acid etching and conditioning with Er,Cr:YSGG laser.	20 deciduous canines were cut in half, half of the samples were prepared with 37% orthophosphoric acid and half with Er,Cr:YSGG laser and then a microtensile test was performed.	The mean SBS was significantly higher in the acid-etched group (24.62 MPa) than in the laser group (18.55 MPa)	The Er,Cr:YSGG laser cannot replace traditional acid etching in primary teeth.
Sungurtekin-Ekci and Oztas [65]	To evaluate the effect of Er,Cr:YSGG laser conditioning alone or in combination with acid etching on the shear bond strength of a fissure sealant for primary tooth enamel.	25 primary molars were divided into 5 groups (different laser parameters 2.5 W and 3.5 W, acid only, laser + acid), then the micro shear bond strength was measured.	Acid etching and the 3.5 W laser gave comparable results, but the 2.5 W laser had significantly lower bond strength; the best result was achieved by 3.5 W + acid.	A 3.5 W laser can be an alternative to acid, but the weaker parameters (2.5 W) are insufficient to obtain good adhesion.
Koyuturk [50]	To investigate the effect of Er:YAG laser on SBS of two self-etching systems for carious dentin in primary teeth.	Ninety primary molars with healthy and carious dentin were prepared using an Er:YAG laser or a drill, two different adhesive systems were used (Clearfil S3, Xeno V) and then microtensile test and SEM analysis were performed.	The highest strength was obtained in the Clearfil S3 + laser group on healthy dentin (24.57 MPa), the lowest in the Xeno V + laser group on healthy dentin (11.01 MPa); Clearfil S3 generally gave better results than Xeno V.	Er:YAG may improve the bonding of some adhesive systems (Clearfil S3), but the effectiveness depends on the type of system used.
Paryab [51]	Evaluation of the effect of two Er:YAG laser energy levels on SBS composite for primary dentin compared to bur preparation using total-etch and self-etch systems.	60 primary molars were divided into 3 groups (drill, 300 mJ laser, 400 mJ laser) and subgroups with total-etch (Single Bond) and self-etch (Clearfil SE Bond) systems; after thermocycling, SBS was measured.	The highest strength was achieved in the drill + Clearfil SE Bond group, the lowest in the 400 mJ laser + Clearfil SE Bond group; the differences between the groups were not statistically significant	Both adhesive systems perform well enough on primary dentin prepared with a 300 and 400 mJ laser, making the laser an alternative to bur preparation.
Memarpour [52]	The study aimed to assess how Er:YAG laser preparation affects the shear bond strength (SBS) and micromorphology of a self-adhering flowable composite when applied to primary tooth enamel and dentin, compared to silicon carbide paper (SiC) preparation.	120 extracted primary canine teeth, divided into enamel and dentin groups. Each group was subdivided based on surface preparation method (SiC or laser) and restorative material: Premise Flowable, Vertise Flow and Vertise Flow combined with adhesive (OptiBond All-In-One). Shear bond strength tests and SEM imaging were performed.	Laser pretreatment increased the SBS of Vertise Flow on enamel compared to SiC (*p* < 0.001). However, on dentin, Er:YAG laser did not improve SBS: the SBS of Vertise Flow after laser treatment remained low and was similar to that on SiC-prepared dentin. Using an adhesive with Vertise Flow significantly improved SBS on both enamel and dentin, regardless of surface preparation. SEM analysis showed that laser created a more irregular surface and opened dentinal tubules more effectively than SiC.	Laser pretreatment improved the bond strength of the self-adhering composite on enamel, but not on dentin. In dentin, laser preparation offered no advantage over conventional SiC treatment in terms of SBS.
Flury [53]	Comparison of micromorphology and adhesive properties of dentin in primary teeth prepared with a diamond drill and Er:YAG laser in different settings.	A total of 82 extracted jbi primary molars were used. 18 teeth were used for SEM micromorphology analysis and 64 molars were used for μTBS adhesive strength testing. For micromorphological analysis, teeth were treated using either a diamond bur or Er:YAG laser at four different energy settings. SEM imaging was conducted to evaluate surface characteristics. For bond strength tests, 64 teeth were prepared with adhesive (Clearfil SE) and composite (Clearfil Majesty Esthetic), then tested with μTBS.	The μTBS testing showed no statistically significant differences in bond strength between diamond bur-treated teeth and laser-treated groups (*p* = 0.394).	Er:YAG laser-treated dentin provides bonding strength to resin composite similar to that achieved with conventional bur preparation. However, insufficient laser energy, as applied in Group 2d (50 mJ/35 Hz), resulted in incomplete dentin surface preparation, leading to less favorable bonding performance.
Yildiz [54]	Evaluation of the impact of three different caries removal methods affect the microtensile bond strength of adhesives to caries-affected dentin.	30 extracted primary molars were divided into three groups based on caries removal technique: conventional steel drill, Er:YAG laser and chemomechanical method—Carisolv, then bonded with either etch-and-rinse or self-etch adhesive. Composite was applied and cured. Teeth were cut into 1 mm^2^ sticks for μTBS testing. Fracture types were examined under a microscope and the dentin surface morphology was analyzed using SEM.	Bond strength was significantly lower in laser-treated groups compared to bur and chemomechanical groups, for both adhesives (*p* < 0.05). No significant difference was observed between conventional drill and Carisolv groups. Adhesive type (etch-and-rinse vs. self-etch) did not significantly affect results.	The caries removal method significantly affects bond strength in primary dentin. Bur and chemomechanical methods resulted in better adhesion than Er-YAG laser technique. The choice between self-etch or etch-and-rinse adhesives does not significantly influence bond strength.
Bolukbasi [70]	To compare how five different pulpotomy techniques affect the shear bond strength of two restorative materials (glass hybrid and composite resin) to primary dentin.	240 dentin samples from 120 caries-free primary molars were divided into six groups (*n* = 40) based on the pulpotomy treatment: Control Group Ferric Sulfate Group Biodentine Group Nd:YAG Laser Group Photobiomodulation Group (LLLT) APCP Group Each group was split into two subgroups (*n* = 20) for restoration with: Glass hybrid material or Composite resin. Shear bond strength (SBS) testing and SEM surface analysis were performed.	Nd:YAG laser showed the highest SBS in both materials. Biodentine showed the lowest SBS for glass hybrid. Ferric sulfate caused significantly lower SBS for composite resin. SEM showed open dentin tubules with Nd:YAG and occluded tubules with Biodentine and ferric sulfate. Composite resin had consistently higher SBS than glass hybrid across all groups.	Nd:YAG laser treatment enhances adhesion strength, especially with composite resin. Ferric sulfate negatively impacts composite bonding.
Borsatto [55]	The study aimed to assess how thermocycling and water storage, which simulate long-term oral conditions, influence the shear bond strength of composite resin applied to primary teeth enamel prepared using an Er:YAG laser compared to traditional bur preparation.	48 primary molars were randomly divided into 2 groups: bur-prepared and Er:YAG laser-prepared cavities. All teeth were restored using an etch-and-rinse adhesive and composite resin. Samples were subjected to aging through water storage (24 h to 6 months) and thermocycling (up to 12,000 cycles). Shear bond strength was measured using a universal testing machine and failure modes were analyzed under a stereomicroscope.	The bur-prepared group exhibited the highest initial bond strength (17.45 MPa after 24 h), but its adhesion significantly decreased after prolonged aging (down to 6.88 MPa after 1 month). Er:YAG laser-prepared enamel showed stable adhesion after 1 month (15.05 MPa), but significant reduction (5.51 MPa) occurred after 6 months.	Significant changes in the adhesion of an etch-and-rinse system to Er:YAG laser-prepared primary enamel were observed only following 6 months of water aging and 12,000 thermal cycles.
Wanderley [56]	Evaluation of the effect of Er:YAG laser etching on the shear bond strength (SBS) of an adhesive system.	The study used 48 primary canines. Their surfaces were cleaned. The teeth were divided into four groups: 1. Acid etching only 2. Acid etching with an Er:YAG laser at 60 mJ 3. Acid etching with an Er:YAG laser at 80 mJ 4. Acid etching with an Er:YAG laser at 100 mJ Bonding system was applied to all samples. Filtek Z250 light-cured composite resin blocks were then applied and polymerised. The composite blocks were then loaded until they detached from the teeth. The SBS was measured. Enamel evaluation was performed using SEM.	Samples etched with a laser power of 60 mJ had higher SBS values than samples etched with phosphoric acid only. The higher the laser power, the lower the SBS values. Acid etching results in a smooth surface and etch pattern. Acid etching + laser: uneven surface and etch pattern. Laser etching produced no pattern and increased surface irregularity with higher laser power.	Using an Er:YAG laser as an additional etching agent can increase the shear bond strength (SBS) if the correct parameters are set. The Er:YAG laser affects the morphological structure of enamel.
Moghini [57]	Evaluation of the effect of Er:YAG laser etching on the morphology of the dentin and the strength of the bond (SBS).	The study involved 69 primary canines. The teeth were cleaned. Forty-eight of these were used for the SBS study. Group 1 received phosphoric acid etching, while Groups 2, 3 and 4 received acid etching plus 60, 80 and 100 mJ of Er:YAG laser irradiation, respectively. A bonding agent was applied to each sample, which was then irradiated. Composite-polyester resin blocks were then bonded to the teeth. The material was then subjected to loading to determine the SBS. Twenty-one teeth were evaluated using SEM. Seven groups were formed: 1. acid etching, 2. acid etching + laser, 3. acid etching + laser, 4. acid etching + laser, 5. laser etching, 6. laser etching, 7. laser etching.	The acid-only etching group achieved the highest SBS values. The laser-etching groups had lower values. Regardless of power, laser etching produced similar SBS values. Acid etching alone creates a regular dentin surface. Laser etching alone creates an irregular dentin surface. Acid and laser etching together create an irregular surface with open channels.	Using a laser to etch dentin does not improve SBS values. Using a laser in conjunction with etching creates an irregular dentin surface, which may affect the holding strength of composite restorations.
Kattan [66]	Assessment of dentin SBS following exposure to an Er,Cr:YSGG laser.	The study used forty primary teeth. The surfaces of the teeth were cleaned. The control group comprised: Ten teeth were etched with phosphoric acid and thirty with an Er,Cr:YSGG laser. Group A of the control group received Rely-X ARC, while groups B, C and D received Rely-X Unicem, Rely-X ARC and glass ionomer cement (GIC), respectively. The samples were loaded and the SBS was measured.	The control group had the highest SBS values. The SBS values in the laser groups B and C were comparable. D group had lower SBS values than B and C.	The highest bond strength is achieved through classical phosphoric acid etching. Treating dentin with an Er,Cr:YSGG laser does not increase the holding strength of the material.
Felemban [58]	Comparison of the bond interface quality between restorations placed after bur preparation and Er:YAG laser preparation.	80 carious primary molars (class I caries) from 40 patients (9–12 years old) treated in one quadrant by bur preparation and in the other by Er:Yag (2940 nm) laser preparation (treatment performed using rubber dam), after 1 year all were assessed clinically and radiographically, 20 teeth were extracted and after preparation assessed using SEM (integration of the adhesive with enamel wall, dentin wall and dentin floor)	No statistically significant difference in bond interface quality of the restorations placed in cavities prepared by bur caries removal or laser method.	The bond interface quality of class I restorations placed in primary molars after Er:YAG laser preparation and bur preparation performed similarly.
Oznurhan [67]	Analysis of the resin-dentin interface in primary teeth after laser or bur cavity preparation.	The V class cavities were prepared in 20 extracted primary molars (10 by bur and 10 by Er,Cr:YSGG laser). Later palatal/lingual cavities were restored following acid-etching and buccal without acid-etching, creating four groups: G1: Er,Cr:YSGG laser G2: Er,Cr:YSGG laser and acid etching G3: Bur G4: Bur and acid etching Then teeth were sectioned and immersed in ammoniacal silver nitrate solution for 24 h in a dark chamber. The resin–dentin interface was assessed by SEM and ion analysis was performed with SEM-energy-dispersive X-ray spectroscopy (EDX).	Surface structure: G1, G2 wavy; G3, G4 smooth. Microcracks: G1, G2. Dentin tubules exposure: G1, G2. Gaps and smear layer: G1, G2 no smear layer, gaps; G3 smear layer and gaps; G4 none. Resin tags: broken in G1, G2 cavities, increased in G2.	For better adhesion after laser preparation including acid-etching is recommended.
Scatena [59]	In vitro assessment of the influence of Er:YAG laser irradiation distance on the shear strength of the adhesive bond.	60 exfoliated/extracted primary molars (embedded in acrylic resin and mechanically treated to expose a flat dentin surface) were divided into 6 groups (*n* = 10): control (etched with 37% phosphoric acid) and rest irradiated (80 mJ, 2 Hz) at different irradiation distances (11, 12, 16, 17, 20 mm), then acid-etched. Later Single bond (adhesive agent) was applied, then resin cylinders (Filtek Z250) were prepared. The shear bond strength were tested by a universal testing machine (0.5 mm/min).	The mean shear bond strengths (MPa) were as follows: Control 7.32 ± 3.83 11 mm 5.07 ± 2.62 12 mm 6.49 ± 1.64 16 mm 7.71 ± 0.66 17 mm 7.33 ± 0.02 20 mm 9.65 ± 2.41 Statistically significant differences were found between 11 mm and 16 mm groups; 11 mm and 20 mm groups	Increasing the laser irradiation distance enhanced the shear bond strength to primary dentin. Er:YAG laser treatment combined with acid etching at a 20 mm distance shows superior adhesive resistance compared to acid etching alone
Nisar [71]	Evaluation of disinfection of caries-effected dentin using KTP laser, Er,YAG laser and other methods on the shear bond strength of adhesive resin.	50 primary molar teeth were qualified by radiological and physical (dental explorer, staining with Caries Detector and visually examined) and divided into 5 groups. Laser conditioning was employed: For KTP laser- 4 times in the row 532 nm applied for 10 s with 5 s waiting period for 1 min at 1 W energy output, 10 t pulsed mode and focal distance 1 mm. Er YAG laser was executed with output 1.2 W, pulse repetition 10 Hz, 2940 nm wavelength, 18.9 J/cm^2^	Caries affected dentin when disinfected with Er YAG have high SBS value. Eradicating carious dentine with Er YAG provided eradication of smear layer and formed resin tags allowing adhesive to reach surface with high roughness leading to high SBS values. KTP laser as cavity disinfectant provoked surface melting and demolished dentinal tubules making them incompetent to form hybrid layers, decreasing SBS values.	Er YAG laser can be implemented as cavity disinfectant in primary teeth reliably as it demonstrated better shear bond strength without negating adhesive binding capacity of used resins.
Kiomarsi [68]	To assess microshear bond strength of a self-adhering flowable composite compared with conventional composite, requiring adhesive and etching procedures to primary enamel treated with graphite disc and laser irradiation.	72 primary teeth was used in the study, being tested no longer than 3 months after exfoliation/extraction. Smooth enamel surface was achieved after treating tooth with grit disc with silicone carbide particles. Then half of the teeth (36) were treated with Er,Cr YSSG laser with wavelength of 2780 nm, 20 Hz frequency, 1.5 W power, 60% water and 60% air cooling with 1 mm distance and sweeping motion, 60 μs pulse duration. Then 2 different composites were applied to laser and non-laser (control) group (self-adhering-VF and one requiring etching and adhesive procedure-PF)	µSBS of self-adhering composites is increased after laser surface treatment of enamel surface of primary teeth. (13.60 ± 5.47) vs. (5.89 ± 2.42) No significant difference was observed when comparing conventional composite µSBS values applied on laser treated enamel vs. non-treated samples (13.18 ± 3.45) vs. (16.06 ± 3.52)	Conventional flowable composites presented higher µSBS values than self-adhering composites for primary teeth. Laser treatment of enamel surface increases µSBS value of self-adhering composites when compared to non-laser treated samples
Bahrololoomi [60]	To evaluate effect of different concentrations of NaOCl on shear bond strength of composite resin to dentin prepared with bur and laser	48 extracted primary human molars with sound buccal and lingual surfaces were used in the study. Specimens were prepared by root sectioning and crowns were divided into buccal and lingual parts, overall 96 specimens were assessed. 12 of them were assessed for SEM scan. Remaining specimens were divided into 2 groups *n* = 42 to be prepared with laser or bur. Then each of those groups were divided into 3: without sodium hypochlorite, with 2,5% and with 5.25% sodium hypochlorite. Laser used for preparation was Er:YAG laser (Fotona, Fidelis Plus III, Slovenia), with 2.94 μm wavelength, 200 mJ energy, pulse repetition rate of 10 Hz and micro-short pulse (MSP) along with air-water cooling of 7 mL/min, in non-contact mode from 17 mm distance. Then all surfaces were etched and NaOCl was applied to specimens and after that bond and composite were applied and after 1000 thermocycles bond strength was calculated in MPa using instron testing machine	Shear bond strength to dentin in laser treated group was a little, but not significantly higher compared to bur group. NaOCl application increased SBS in both groups but also without any significant differences.	Difference between laser and bur group was not statistically significant, although laser treated surface of primary teeth presented increase in SBS. This is probably connected to morphological properties of laser treated surface compared to bur prepared surface. The structural difference in primary teeth dentin with permanent ones is probably the reason for this insignificant difference, despite the complete removal of collagen. Primary teeth are less calcified than permanent teeth, and contain more water and organic substances. The dentinal tubules are less aligned and intertubular dentin contains more water than peritubular dentin. Therefore, primary teeth may need different application time and concentration of sodium hypochlorite in order to improve the bond strength compared to permanent teeth; thus the need for more research in this field.

**Table 2:** Detailed characteristics of included studies.

Study	Laser Type and Parameters	Sample Type	Adhesive/Restorative Material	Shear/Tensile Bond Strength	Morphological Analysis
Ersan [61]	Er, Cr:YSGG Device: Waterlase iPlus (Biolase, San Clemente, CA, USA) Wavelength: 2780 nm Pulse time: 140 μs Tip: sapphire, diameter 600 μm, length 6 mm Power: 0.75 W Cooling: 15% water, 15% air Frequency: 20 Hz Distance: 1–2 mm Operating mode: 5 applications of 10 s each, with 5 s breaks	Enamel	pit and fissure sealant ClinPro Sealant (3M ESPE, St. Paul, MN, USA)	Laser + CPP–ACP: 18.23 ± 5.9 MPa Laser: 18.29 ± 7.6 MPa NaOCl: 6.42 ± 2.5 MPa NaOCl + CPP–ACP: 6.23 ± 3.2 MPa CPP–ACP: 15.61 ± 7.9 Mpa Control: 16.09 ± 6.8 MPa	In the Laser, Laser + CPP–ACP, CPP–ACP, and control groups, cohesive and mixed fractures predominated. In the NaOCl and NaOCl + CPP–ACP groups, adhesive fractures predominated.
Chikkanarasaiah and Hrishida [62]	Er, Cr:YSGG Wavelength: 2780 nm Energy: 0.5 W, 25 mJ/pulse Energy density: 9 J/cm^2^ Tip: sapphire, distance: 2 mm Scanning mode	Dentin	Conventional glass ionomer cement—GC Fuji IX (GC Corporation, Tokyo, Japan)	Control: 3.033 ± 0.065 MPa Laser: 3.381 ± 0.088 MPa	The laser removes the smear layer and opens the dentinal tubules, creating an irregular, rough surface that promotes adhesion.
Borsatto [46]	Er:YAG Device: KaVo Key Laser 2 (KaVo, Biberach, Germany) Wavelength: 2.94 μm Pulse energy: 80 mJ Frequency: 2 Hz Operating mode: defocused mode Exposure time: 30 s Focal distance: 17 mm Water jet: 1.5 mL/min Beam spot diameter: 0.63 mm Beam area: ~0.312 mm^2^	Enamel	Pit and fissure sealant FluroShield (Caulk-Dentsply, Milford, DE, USA)	37% phosphoric acid: 10.80 ± 3.28 MPa Er:YAG + 37% phosphoric acid: 12.85 ± 2.14 MPa Er:YAG: 4.17 ± 2.31 MPa	Irregular, non-uniform etch pattern, excessive subsurface cracking, micropores partially submerged by fused/resolidified enamel; this resulted in a higher rate of adhesive fractures.
Kotb [69]	Diode Laser Wavelength: 980 nm Power: 1 W Operation mode: continuous Fiber diameter: 300 μm Distance: ~1 mm Traverse speed: 1 mm/s Time: 10 s Irradiation field diameter: 1 mm (area ~0.785 mm^2^)	Enamel Dentin	Activa Bioactive Restorative material (Pulpdent, Watertown, MA, USA) Filtek Z350XT (3M ESPE, St. Paul, MN, USA)—nanohybrid composite All-Bond Universal Adhesive (Bisco, Schaumburg, IL, USA)—universal bonding system	Enamel/Activa Laser = 19.68 ± 3.65 Mpa control = 24.70 ± 2.92 Mpa Enamel/Composite Laser = 18.44 ± 7.49 MPa control = 20.25 ± 3.68 Mpa Dentin/Activa Laser = 13.61 ± 3.07 MPa control = 17.88 ± 3.46 Mpa Dentin/Composite Laser = 19.74 ± 3.64 Mpa control = 17.63 ± 2.63 MPa	The highest number of adhesive fractures occurred in the Dentin/Activa/No Laser group (76.9%) and the Dentin/Composite/Laser group (75%). The lowest number of adhesive fractures occurred in the Enamel/Activa/Laser group (18%).
Babu [47]	Er:YAG Wavelength: 2940 nm Max output power: 10 W Pulse energy: 200 mJ Pulse repetition rate: 10 Hz Pulse duration: 450 µs Spot size: 0.785 mm^2^ Distance: 8–12 mm, non-contact mode Preparation parameters: enamel—4 W, 10 Hz, 60% air-water; dentin—3 W, 60% air, 30% water Er,Cr:YSGG Wavelength: 2780 nm Max output power: 10 W Max pulse energy: 600 mJ Pulse repetition rate: 5–100 Hz Pulse duration: 600–700 µs Spot size: 0.442 mm^2^ Distance: 8–10 mm, non-contact mode Preparation parameters: enamel—4 W, 15 Hz, 60% air-water; dentin—3 W, 60% air, 30% water	Enamel dentin	Composite Filtek™ 350 XT (3M ESPE, St. Paul, MN, USA) Adper™ Single Bond 2 Adhesive (3M ESPE, St. Paul, MN, USA)	Bur + acid: 17.562 ± 0.810 MPa Er:YAG+acid: 15.928 ± 0.415 MPa Er,Cr:YSGG + acid: 14.964 ± 0.566 MPa Er:YAG without acid: 11.833 ± 0.533 MPa Er,Cr:YSGG without acid: 11.187 ± 0.517 MPa	The laser creates an irregular, scaly surface with open dentinal tubules and no smear layer. This may include a remelted layer, microcracks, adhered collagen fibers and porous layers of recrystallized minerals after microblasting.
AlHumaid [63]	Er,Cr:YSGG Wavelength: 2780 nm Fiber diameter: 600 μm Power: 2.5 W or 3.5 W Pulse time: 140 μs Frequency: 20 Hz Pulse energy: 75 mJ/pulse Operating mode: non-contact, 1 mm distance Irradiation time: 15 s Air/water cooling: 70% air/60% water (2.5 W) and 80% air/70% water (3.5 W)	enamel	Pit and fissure sealant ClinPro™ Sealant (3M ESPE, St. Paul, MN, USA) Composite Filtek Z350 (3M ESPE, St. Paul, MN, USA)	3.5 W laser + acid = 15.57 ± 3.71 MPa 2.5 W laser + acid = 14.18 ± 2.83 MPa 3.5 W laser = 14.78 ± 1.71 MPa 2.5 W laser = 9.66 ± 2.20 MPa Acid = 14.63 ± 3.73 MPa	Er,Cr:YSGG causes micro-explosions in the enamel, leading to macro- and microscopic irregularities, removal of water and mineral components.
Wang [48]	Er:YAG Wavelength: 2940 nm Distance: 1 mm Beam diameter: 0.6 mm Time: 10 s (grid scanning mode) Cooling: 60% water, 40% air/steam Energy: 50, 100, 150, 200, 250, 300 mJ (at 10 Hz) Frequency: 5, 10, 15, 20, 25, 30 Hz (at 100 mJ)	Dentin	Composite Filtek Z350 (3M ESPE, St. Paul, MN, USA)	Control 11.06 ± 2.10 MPa Acid etching 13.74 ± 1.73 MPa 50 mJ 17.74 ± 2.63 MPa 100 mJ 18.07 ± 2.03 MPa 150 mJ 18.11 ± 2.15 MPa 200 mJ 17.56 ± 2.54 MPa 250 mJ 13.39 ± 2.41 MPa 300 mJ 11.27 ± 2.30 MPa	Er:YAG 50–200 mJ/5–20 Hz: open dentinal tubules, absent smear layer, irregular surface with fish-scale-like (lamellar) pattern, protruding areas of peritubular dentin; at 150–200 mJ, fine cracks are visible. Er:YAG > 200 mJ > 20 Hz: loss of lamellar pattern, blurred tubule boundaries, enlarged cracks, collapse of dentin structure, localized charring.
Bahrololoomi [49]	Er:YAG Wavelength: 2940 nm Energy: Enamel: 300 mJ, 10 Hz Dentin: 200 mJ, 10 Hz Operating mode: non-contact Distance: 17 mm Cooling: water 7 mL/min	Enamel Dentin	Adhesive Adper™ Single Bond 2 (3M ESPE, St. Paul, MN, USA) Composite Filtek™ Z250 (3M ESPE, St. Paul, MN, USA)	Drill+acid 14.53 ± 3.53 MPa Laser + acid 10.69 ± 2.08 MPa Laser without acid 3.53 ± 0.89 MPa	Possible closure of the dentinal tubule orifices and fusion of collagen fibers
Malekafzali [64]	Er,Cr:YSGG Wavelength: 2780 nm Tip diameter: 600 μm Power: 1.5 W Energy: 75 mJ/pulse Pulse duration: 140 μs Frequency: 20 Hz Total energy delivered: 15 J Scanning speed: 1 mm/s Distance from surface: 1–2 mm Irradiation pattern: vertical and horizontal Cooling: 55% water, 65% air, water flow 12 mL/min	enamel	adhesive: Single Bond (3M ESPE, St. Paul, MN, USA) Composite: Z100 (3M ESPE, St. Paul, MN, USA)	Control = 24.62 ± 5.56 MPalaser = 18.55 ± 6.41 MPa	No data
Sungurtekin-Ekci and Oztas [65]	Er,Cr:YSGG Wavelength: 2780 nm Pulse duration: 140–200 μs Frequency: 20 Hz Power: 2.5 W or 3.5 W Operating mode: non-contact, distance 1 mm from the surface Tip: sapphire, diameter 600 μm, length 6 mm Spot area: 0.442 mm^2^ Cooling: 90% air, 80% water Exposure time: 10–15 s	Enamel	ClinPro™ (3M ESPE, St. Paul, MN, USA)—resin fissure sealant Composite Filtek™ Z250 (Shade A3.5, 3M ESPE, St. Paul, MN, USA)	Control = 12.18 ± 3.95 MPa Laser 2.5 W = 8.30 ± 1.84 MPa Laser 3.5 W = 11.57 ± 3.27 MPa Laser 2.5 W + acid = 12.67 ± 4.51 MPa Laser 3.5 W + acid = 13.04 ± 3.62 MPa	SEM revealed that in the laser groups the surface was irregular, with microcracks and depressions.
Koyuturk [50]	Er:YAG Wavelength: 2940 nm Pulse energy: 200 mJ Frequency: 20 Hz Power: 4.0 W Pulse duration: 100 μs Tip: sapphire, diameter 1.3 mm, length 8 mm	dentine	Adhesives: Clearfil S3 Bond (Kuraray, Osaka, Japan), Xeno V Bond (Dentsply Detrey, Konstanz, Germany) Dyract Extra (Dentsply Detrey, Konstanz, Germany)—compomer	Sound enamel: Bur + Clearfil S3 = 20.32 ± 7.54 MPa Laser + Clearfil S3 = 24.57 ± 7.27 MPa Bur + Xeno V = 12.10 ± 4.80 MPa Laser + Xeno V = 11.02 ± 3.90 MPa Caries: Bur + Clearfil S3 = 15.51 ± 5.49 MPa Laser + Clearfil S3 = 19.30 ± 6.12 MPa Bur + Xeno V = 12.40 ± 5.04 MPa Laser + Xeno V = 13.12 ± 4.33 MPa	For Clearfil S3—in sound and caries-affected dentine after laser and drilling, the hybrid layer was continuous, well-adhered and had numerous, long tags. For Xeno V—also a continuous layer, but shorter and less distinct tags compared to Clearfil S3.
Paryab [51]	Er:YAG Wavelength: 2940 nm Pulse duration: 450 μs Focal spot size: 0.785 mm^2^ Cooling: air/water Distance: 0.8–1.2 cm, non-contact, defocused mode Power: 3 or 4 W Energy: 300 or 400 mJ Frequency: 10 Hz	Dentine	Adhesives: Single Bond (3M ESPE, St. Paul, MN, USA) or Clearfil SE Bond (Kuraray, Osaka, Japan) Composite Filtek Z250 (3M ESPE, St. Paul, MN, USA)	Drill + Single Bond = 6.94 ± 2.12 MPa Drill + Clearfil SE = 9.00 ± 2.47 MPa Laser 300 mJ + Single Bond = 6.28 ± 2.58 MPa Laser 300 mJ + Clearfil SE = 6.42 ± 3.90 MPa Laser 400 mJ + Single Bond = 6.00 ± 3.16 MPa Laser 400 mJ + Clearfil SE = 5.62 ± 2.99	The laser prepares a dentin surface with open tubules, no smear layer and no signs of charring within the parameters used.
Memarpour [52]	Laser type: Er:YAG Wavelength: not reported Time: not reported Distance: 1 mm Power: 1.20 W Power density: not reported Beam diameter: 0.8 mm Surface area: not reported Fluence: not reported Frequency: 10 Hz Pulse duration: 100 μs Cooling: Water 8, Air 4 Energy: 120 mJ	Enamel and dentin	One-step self-etch adhesive: OptiBond All-In-One (Kerr, Orange, CA, USA) Conventional flowable composite: Premise Flowable (Kerr, Orange, CA, USA) Self-adhering flowable composite: Vertise Flow (Kerr, Orange, CA, USA) Group combinations: Adhesive + Premise Flowable Adhesive + Vertise Flow Vertise Flow alone	Enamel—SiC pretreatment Adhesive + Premise Flowable: 13.06 ± 2.36 MPa Adhesive + Vertise Flow: 15.05 ± 2.12 MPa Vertise Flow: 9.29 ± 1.56 MPa Enamel—Laser pretreatment Adhesive + Premise Flowable: 13.90 ± 3.16 MPa Adhesive + Vertise Flow: 16.16 ± 2.76 MPa Vertise Flow: 14.84 ± 1.32 MPa Dentin—SiC pretreatment Adhesive + Premise Flowable: 17.41 ± 1.20 MPa Adhesive + Vertise Flow: 16.89 ± 1.05 MPa Vertise Flow: 12.17 ± 1.31 MPa Dentin—Laser pretreatment Adhesive + Premise Flowable: 17.65 ± 1.25 MPa Adhesive + Vertise Flow: 13.93 ± 0.97 MPa Vertise Flow: 12.09 ± 1.26 MPa	Enamel: SiC: smear layer present. Laser: no smear layer, rougher surface. Adhesive after laser: more mineral loss in interprismatic areas, prism heads exposed compared to SiC. Vertise Flow: mild etching, more marked after laser than SiC. Dentin: SiC: smear layer covered most tubules. Laser: open tubules, irregular surface, no smear layer, no thermal damage. Adhesive on laser: slight roughness increase; on SiC: partial smear layer removal incomplete, partial exposure of the tubules. Vertise Flow: fewer open tubules than laser alone, more surface irregularities after laser; partial smear layer removal and fewer open dentinal tubules after SiC.
Flury [53]	Laser type: Er:YAG Wavelength: 2.94 μm Group 2a Energy: Preparation: 200 mJ Finishing: 100 mJ Frequency: Preparation: 25 Hz Finishing: 35 Hz Power: Preparation: 5.0 W Finishing: 3.5 W Pulse duration: ~450 μs Distance: 2 mm Beam diameter: 1 mm Cooling: water-cooled Time: >300 s (preparation + finishing) Power density: no data Fluence: no data Surface area: no data Group 2b: Energy: 400 mJ Frequency: 20 Hz Power: 8.0 W Pulse duration: ~450 μs Distance: 2 mm Beam diameter: 1 mm Cooling: water-cooled Time: 30–40 s Power density: no data Fluence: no data Surface area: no data Group 2c: Energy: 100 mJ Frequency: 35 Hz Power: 3.5 W Pulse duration: ~450 μs Distance: 2 mm Beam diameter: 1 mm Cooling: water-cooled Time: 60–90 s Power density: no data Fluence: no data Surface area: no data Group 2d: Energy: 50 mJ Frequency: 35 Hz Power: 1.75 W Pulse duration: ~450 μs Distance: 2 mm Beam diameter: 1 mm Cooling: water-cooled Time: 100–130 s Power density: no data Fluence: no data Surface area: no data	Dentin	Adhesive: Clearfil SE (self-etch system)—Kuraray, Osaka, Japan Restorative composite: Clearfil Majesty Esthetic—(Kuraray, Osaka, Japan)	μTBS: Group 1a: not measured Group 1b (Grinding + 40 μm diamond bur): 24.8 ± 6.6 MPa Group 2a: not measured Group 2b (Grinding + Er:YAG laser): 26.1 ± 4.3 MPa Group 2c (Grinding + Er:YAG laser): 22.2 ± 8.6 MPa Group 2d (Grinding + Er:YAG laser): 23.9 ± 6.1 MPa No significant differences between groups (*p* = 0.394)	SEM observations: Bur-treated dentin: smooth surface with a uniform smear layer, no open tubules. Laser-treated dentin: no smear layer, open dentinal tubules and scaly, irregular surface. Group 2d: Incomplete ablation; parts of the SiC-ground surface remained visible. No signs of thermal damage.
Yildiz [54]	Laser type: Er:YAG Wavelength: 2.94 μm Time: no data Distance: 1 mm Power: 3.5 W Power density: no data Beam diameter: 1 mm Surface area: no data Fluence: 44 J/cm^2^ Frequency: 10 Hz Pulse duration: 300 μs Cooling: continuous air and water spray Energy: no data	Dentin	Adhesive systems: Adper Single Bond 2 (etch-and-rinse; 3M ESPE, St. Paul, MN, USA) G-Bond (one-step self-etch; GC Corporation, Tokyo, Japan) Restorative material: Filtek Z250 (3M ESPE, St. Paul, MN, USA) Gradia Direct (GC Corporation, Tokyo, Japan)	Microtensile bond strength (μTBS): Rotary bur + Etch-and-rinse: 20.77 ± 5.64 MPa Rotary bur + Self-etch: 21.05 ± 5.19 MPa Er:YAG laser + Etch-and-rinse: 15.70 ± 5.87 MPa Er:YAG laser + Self-etch: 16.51 ± 4.91 MPa Carisolv + Etch-and-rinse: 19.29 ± 4.30 MPa Carisolv + Self-etch: 19.37 ± 3.56 MPa	SEM observations: Bur-treated dentin: A smear layer was present, partially blocking the visibility of the dentinal tubules. Smear plugs were present, filling some tubule openings. The overall surface appeared irregular. Laser-treated dentin: No smear layer Dentinal tubules were open and distributed across a scaly and irregular surface. Intertubular dentin was more ablated compared to peritubular dentin. Visible collagen fibrils Microretentive pattern Carisolv-treated dentin: A smear layer was present, with plugs occluding parts of the tubule entrances. Some open tubules visible
Bolukbasi [70]	Laser type: Nd:YAG Wavelength: 1064 nm Time: 10 s Distance: no data Power: 2 W Power density: no data Beam diameter: 300 μm Surface area: no data Fluence: no data Frequency: 20 Hz Pulse duration: no data Cooling: no data Energy: 100 mJ	Dentin	Composite resin: Filtek Z550 Posterior Restorative (3M ESPE, St. Paul, MN, USA) Glass hybrid cement: GC Equia Forte (GC Corporation, Tokyo, Japan) Adhesive system for composite: Single Bond Universal (3M ESPE, St. Paul, MN, USA)	SBS results Nd:YAG laser—Composite resin: 13.79 ± 1.24 MPa Nd:YAG laser—Glass hybrid: 7.58 ± 0.60 MPa APCP—Composite resin: 13.35 ± 0.65 MPa APCP—Glass hybrid: 7.54 ± 0.99 MPa Control—Composite resin: 12.85 ± 1.34 MPa Control—Glass hybrid: 7.41 ± 0.81 MPa Biodentine—Composite resin: 12.60 ± 1.17 MPa Biodentine^®^—Glass hybrid: 6.70 ± 0.91 MPa Photobiomodulation—Composite resin: 12.14 ± 1.47 MPa Photobiomodulation—Glass hybrid: 6.92 ± 0.77 MPa Ferric sulfate—Composite resin: 10.17 ± 1.45 MPa Ferric sulfate—Glass hybrid: 7.21 ± 0.76 MPa	SEM observations: Nd:YAG laser: Smear layer removed, partially opened dentinal tubules Biodentine: Tubules occluded by material Ferric sulfate: Some tubules occluded and some open Photobiomodulation: similar to control group, smear layer remained, tubules not clearly opened APCP: Similarly to control group
Borsatto [55]	Laser type: Er:YAG Wavelength: 2.94 μm Time: 20 s Distance: 12 mm Power: no data Power density: Beam diameter: 0.63 mm Surface area: no data Fluence: no data Frequency: 2 Hz Pulse duration: no data Cooling: fine water mist during irradiation Energy: 250 mJ	Enamel	Adhesive system: Single Bond 2 Adper (3M ESPE, St. Paul, MN, USA)—etch-and-rinse type adhesive Restorative material: Filtek Z250 light-cured composite resin (3M ESPE, St. Paul, MN, USA)	SBS: AI (Bur, 24 h Water Storage/0 Thermal Cycles): 17.45 ± 2.03 MPa AII (Bur, 7 d WS/500 TCs): 16.38 ± 1.49 MPa AIII (Bur, 1 month WS/2000 TCs): 6.88 ± 0.66 MPa AIV (Bur, 6 months WS/12,000 TCs): 7.77 ± 1.53 MPa BI (Laser, 24 hWS/0 TCs): 12.32 ± 0.99 MPa BII (Laser, 7 d WS/500 TCs): 15.37 ± 2.24 MPa BIII (Laser, 1 month WS/2000 TCs): 15.05 ± 2.01 MPa BIV (Laser, 6 months WS/12,000 TCs): 5.51 ± 1.01 MPa	The fractured specimens were examined under a stereomicroscope at 40× magnification to classify the type of failure. SEM analysis was not used in this study. In the groups where enamel was prepared with the Er:YAG laser, failures were predominantly adhesive failures. As the duration of water storage and the number of thermal cycles increased, the proportion of mixed failures became higher. Importantly, no cohesive fractures were detected in any of the groups.
Wanderley [56]	Laser type: Er:YAG Wave length: 2.94 μm Energy: 60, 80, 100 mJ Distance: 17 mm Time: 1 min and 30 sec Frequency: 2 Hz	Enamel	Hybrid light-curing composite resin (Filtek Z250, 3M ESPE, St. Paul, MN, USA)	Control—35% phosphoric acid= 14.28 (±3.24) MPa Er:YAG laser 60 mJ/2 Hz + acid= 18.48 (±4.58) MPa Er:YAG laser 80 mJ/2 Hz + acid= 17.82 (±4.38) MPa Er:YAG laser 100 mJ/2 Hz + acid= 16.59 (±5.40) MPa	Control- even surface, type II etching pattern, demineralized interprismatic part, surface microporosity Er:YAG 60 mJ/2 HZ- irradiation: uneven surface, no etching pattern, after acid etching: slightly micro-rough surface + few crevices and melting points, type I etching pattern + demineralization of the prism centers Er:YAG 80 mJ/2 HZ- Laser irradiation: irregular surface, gaps, small areas of re-melting, unclear etching pattern, acid etching: flaking surface, gaps, small areas of re-melting Er:YAG 100 mJ/2 HZ- Laser irradiation: fractal image, multiple fissures, no etching pattern. Acid etching: microrough surface, uneven topography, multiple adhesions.
Moghini [57]	Laser type: Er:YAG Wave length: 2.94 μm Energy: 60, 80, 100 mJ Distance: 17 mm Time: 1 min and 30 sec Frequency: 2 Hz	Dentin	Hybrid light-curing composite resin (Filtek Z250, 3M ESPE, St. Paul, MN, USA)	Control—35% phosphoric acid= 17.89 (4.75) MPa Er:YAG laser 60 mJ/2 Hz + acid= 12.34 (4.85) MPa Er:YAG laser 80 mJ/2 Hz + acid= 10.30 (3.67) MPa Er:YAG laser 100 mJ/2 Hz + acid= 10.41 (4.20) MPa	Control- regular topography, demineralized peritubular dentin, open and widened tubule entrances Er:YAG 60 mJ/2 HZ- Laser irradiation: uneven tissue surfaces, few open tubules, after acid etching, uneven tissue, more open tubules, absent smear layer, cratered intertubular dentin Er:YAG 80 mJ/2 HZ- Irradiation: irregular surface—craters, fissures, cracks, few open channels, some with a smear layer, after etching: more regular, flaky surface, wider channel openings Er:YAG 100 mJ/2 HZ- irradiation: uneven and flaking surface, noticeable fissures, cracks, open tubules. After etching: irregular, flaking surface, many cracks over the entire surface, numerous open tubules, noticeable peritubular dentin, irregular intertubular dentin.
Kattan [66]	Laser type: Er,Cr:YSGG Wave length: 2780 nm Pulse duration: 140μs Frequency: 20 Hz	Dentin	-Rely X ARC (3M ESPE, St. Paul, MN, USA) -Rely X Unicem (AplicapTM/MaxicapTM,3M ESPE, St. Paul, MN, USA) -GIC: Ketac Cem Aplicap (3M, Seefeld, Germany) ESPE, St. Paul, MN, USA)	Group (Rely-X ARC) A - 25.38 Mpa Group B (ECL-RelyX-Uni) - 20.47 Mpa Group C (ECL-RelyX-ARC) - 21.65 Mpa Group D (ECL-GIC) - 15.91 Mpa	SEM was not performed.
Felemban [58]	Er:YAG laser Wavelength: 2940 nm, used by the instructions of manufacturer (not included in the paper)	Enamel and dentin	Clearfil Universal Bond Quick system (Kuraray Noritake Dental, Tokyo, Japan) 3MTM FiltekTM Z350 XT Universal Restorative, 3M ESPE, St. Paul, MN, USA	Data not included in the study	Final SEM analysis on 20 teeth-one tooth prepared by bur, other by laser (10 subjects: one tooth prepared by bur and one by laser at each subject) -all 20 restorations were integrated with enamel and dentin walls (prepared conventionally or with laser) -adhesive integration with the dentin floor: 6 subjects had both their restorations fully integrated, 4 restorations were not integrated (2 subjects had restoration prepared conventionally not integrated, while prepared by laser integrated; other 2 subjects had restoration prepared by bur integrated to dentin floor, while prepared by laser were not)
Oznurhan [67]	Erbium, chromium:yttrium–scandium–gallium–garnet (Er,Cr:YSGG) laser Power output: 6.0 W (85–90% air and 80–85% water) for Enamel; 3.5 W (70% air and 65% water) for dentine working distance of 1.5–2 mm. (defocused mode)	Dentin	Single Bond AdperTM 3M ESPE FiltekTM Z250, 3M ESPE, St. Paul, MN, USA	Data not included in the study	SEM analysis: -G1 and G2, the surfaces were wavy -G3 and G4, the surfaces were smooth -Microcracks were observed in some of the lased cavities -G1 and G2 the dentin tubules were exposed, and there were the lack of smear layer and gaps -G3 the smear layer and gaps were observed -G4 no gaps and smear layer -G2 increased resin tags were observed (-G1, a thinner hybrid layer or in some parts, the absence of the hybrid layer was observed). -the hybrid layer was thicker in G2 compared to G4 -some resin tags were broken in cavities that were prepared with laser. SEM-energy-dispersive X-ray spectroscopy (EDX) analysis: in the acid-etched groups (G2, G4) the silver ions were observed in hybrid layer and dentin tubules
Scatena [59]	Er:YAG laser -wavelength 2.94 μm at pulse energy 80 mJ -repetition rate: 2 Hz -laser beam: 0.63 mm -varying irradiation Distance: 11, 12, 16, 17, 20 [mm]	Dentin	Single Bond (3M ESPE, St. Paul, MN, USA) Filtek Z250 (3M ESPE, St. Paul, MN, USA)	SBS MPa: G1 7.32 ± 3.83 (control) G2 5.07 ± 2.62 (11 mm) G3 6.49 ± 1.64 (12 mm) G4 7.71 ± 0.66 (16 mm) G5 7.33 ± 0.02 (17 mm) G6 9.65 ± 2.41 (20 mm)	Data not included in the study
Nisar [71]	KTP Laser: Device: (Smartlite D, Deka, Calenzano Firenze, Italy) Wavelength: 532 nm Energy output: 1 W 10 t pulsed mode Focal distance 1 mm Er:YAG Laser: Device (Nubway, Beijing, China) Wavelength: 2940 nm Energy density: 18.9 J/cm^2^ Pulse duration 150 s Repetition rate: 10 Hz Irrigation 5 mL/min	Caries affected dentin	Adper prime NT Bond (Dentsply Detrey, Konstanz, Germany) Resin composite: Tetric N-Ceram (Ivoclar Vivadent, Schaan, Liechtenstein)	SBS MPa KTP Laser group 8.25 ± 0.41 Er:YAG laser group: 10.23 ± 0.33 Control group (Dentin disinfected with CHX) 8.19 ± 0.73	Samples were characterized under 40× magnification with stereomicroscope to assess fracture mode.
Kiomarsi [68]	Er,Cr:YSGG Device: iPlus Waterlase; Biolase, USA Gold MZ6 tip Frequency: 20 Hz Output power 1.5 W Coolant: 60% water, 60% air Distance: 1 mm to the enamel surface Sweeping motion 60 μs pulse duration (10 s for each surface)	Enamel	Vertise Flow (Kerr, Orange, CA, USA) Premise Flow (Kerr Italia S.r.l., Scafati, Italy) Adper Single Bond 2 (3M ESPE St Paul, MN, USA)	µSBS MPa For Vertise Flow Laser treated samples (13.60 ± 5.47) Non treated samples (5.89 ± 2.42) For Premise Flow (13.18 ± 3.45) Non treated samples (16.06 ± 3.52)	Data not included with the study
Bahrololoomi [60]	Er:Yag laser Device: Fotona, Fidelis Plus III, Slovenia Handpiece: RO2-C-919 Wavelength: 2940 nm Energy: 200 mJ Pulse repetition 10 Hz Water cooling 7 mL/min Non-contact mode within 17 mm distance	Enamel (removed with bur or laser, respectively), dentin	One-Step Plus (Bisco, Schamburg, IL, USA) Bonding Agent AELITE Composite (Bisco, Schamburg, IL, USA)	SBS MPa Bur treated samples 13.56 ± 3.36 (No hypochlorite used) 13.53 ± 3.64 (2,5% hypochlorite used) 14.36 ± 3.64 (5,25% hypochlorite used) Laser treated samples: 13.39 ± 3.1 (No hypochlorite used) 14.63 ± 3.93 (No hypochlorite used) 14.51 ± 4 (No hypochlorite used)	SEM Microscopy with 1000× and 5000× magnification. Surface roughness, presence of smear layer, appearance of dentinal tubules and presence of melting or crack were analyzed.

**Table 3:** Recommended Er:YAG and Er,Cr:YSGG laser parameter ranges for enamel and dentin in primary teeth, synthesized from the included studies.

Tooth Tissue	Laser Type	Energy (mJ)	Power (W)	Frequency (Hz)	Cooling	Observed Effect on Bond Strength	References
Enamel	Er:YAG	120–200	1.2–2.0	10–20	Air–water	Optimal micro-retention; with acid etch → SBS comparable/superior to bur; ≥250 mJ ↓ adhesion	[51,53,60,61,68,70]
Enamel	Er,Cr:YSGG	150–175	3.0–3.5	20	Air–water	Clean, micro-rough surfaces; SBS ≈ acid etch; ~3.5 W best; >4 W ↓ adhesion	[68,70]
Dentin	Er:YAG	80–150	0.8–1.5	5–15	Air–water	Highest µTBS/SBS; smear removal + open tubules; ≥250–300 mJ → microcracks, thermal change ↓ bonding	[53,55,58,62,65]
Dentin	Er,Cr:YSGG	125–175	2.5–3.5	20	Air–water	µTBS ↑ esp. with phosphoric acid; ~3.5 W optimal; >4 W → morphological deterioration	[68,70]

**Table 4:** Quality assessment among the included studies.

Author	Q1	Q2	Q3	Q4	Q5	Q6	Q7	Q8	Q9
Ersan [62]	yes	Yes	Yes	no	yes	yes	yes	yes	yes
Chikkanarasaiah [62]	Yes	Yes	Yes	No	Yes	yes	Yes	Yes	yes
Borsatto [46]	Yes	Yes	Yes	No	Yes	yes	Yes	Yes	yes
Kotb [69]	Yes	Yes	Yes	No	Yes	yes	Yes	Yes	yes
Babu [47]	Yes	Yes	Yes	No	Yes	yes	Yes	Yes	yes
Alhumaid [63]	Yes	Yes	Yes	No	Yes	yes	Yes	Yes	yes
Wang [48]	Yes	Yes	Yes	No	Yes	yes	Yes	Yes	yes
Bahrololoomi [49]	Yes	Yes	Yes	No	Yes	yes	Yes	Yes	yes
Malekafzali [64]	Yes	Yes	Yes	No	Yes	yes	Yes	Yes	yes
Sungurtekin-Ekci [65]	Yes	Yes	Yes	No	Yes	yes	Yes	Yes	yes
Koyuturk [50]	Yes	Yes	Yes	No	Yes	yes	Yes	Yes	yes
Paryab [51]	Yes	Yes	Yes	No	Yes	yes	Yes	Yes	yes
Memarpour [52]	Yes	Yes	Yes	No	Yes	yes	Yes	Yes	yes
Flury [53]	Yes	Yes	Yes	No	Yes	yes	Yes	Yes	yes
Yildiz [54]	Yes	Yes	Yes	No	Yes	yes	Yes	Yes	yes
Bolukbasi [70]	Yes	Yes	Yes	No	Yes	yes	Yes	Yes	yes
Borsatto [55]	yes	Yes	Yes	No	Yes	yes	Yes	Yes	yes
Wanderley [56]	Yes	Yes	Yes	No	Yes	yes	Yes	Yes	yes
Monghini [57]	Yes	Yes	Yes	No	Yes	yes	Yes	Yes	yes
Kattan [66]	Yes	Yes	Yes	No	Yes	yes	Yes	Yes	yes
Felemban [58]	Yes	Yes	Yes	No	Yes	yes	Yes	Yes	no
Oznurhan [67]	Yes	Yes	yes	No	Yes	yes	Yes	Yes	no
Scatena [59]	Yes	Yes	Yes	No	Yes	yes	Yes	Yes	yes
Nisar [68]	Yes	Yes	Yes	No	Yes	yes	Yes	Yes	yes
Kiomarsi [68]	Yes	Yes	Yes	No	Yes	yes	Yes	Yes	yes
Bahroloomi [60]	yes	Yes	Yes	No	Yes	yes	Yes	Yes	yes

## Data Availability

No new data were created or analyzed in this study. Data sharing is not applicable to this article.

## Supplementary Materials

The following supporting information can be downloaded at: https://www.mdpi.com/article/10.3390/ma18225212/s1, Table S1. PRISMA 2020 Checklist [72].

## Abbreviations

The following abbreviations are used in this manuscript:

AFM	Atomic Force Microscopy
CHX	Chlorhexidinebio
CPP–ACP	Casein Phosphopeptide–Amorphous Calcium Phosphate
EDX	Energy-Dispersive X-ray Spectroscopy
Er:YAG	Erbium-doped Yttrium Aluminum Garnet laser
Er,Cr:YSGG	Erbium, Chromium-doped Yttrium Scandium Gallium Garnet laser
JBI	Joanna Briggs Institute
KTP	Potassium Titanyl Phosphate laser
MSP	Micro Short Pulse
Nd:YAG	Neodymium-doped Yttrium Aluminum Garnet laser
SBS	Shear Bond Strength
SEM	Scanning Electron Microscopy
µSBS	Microshear Bond Strength
µTBS	Microtensile Bond Strength
WoS	Web of Science
